# Characterising a Custom-Built Radio Frequency PECVD Reactor to Vary the Mechanical Properties of TMDSO Films

**DOI:** 10.3390/molecules26185621

**Published:** 2021-09-16

**Authors:** Racim Radjef, Karyn L. Jarvis, Colin Hall, Andrew Ang, Bronwyn L. Fox, Sally L. McArthur

**Affiliations:** 1 Faculty of Science, Engineering and Technology, Swinburne University of Technology, Melbourne, VIC 3122, Australia; rradjef@swin.edu.au (R.R.); kljarvis@swin.edu.au (K.L.J.); aang@swin.edu.au (A.A.); blfox@swin.edu.au (B.L.F.); 2 Australian Research Council (ARC) Industrial Transformation Training Centre in Surface Engineering for Advanced Materials (SEAM), Swinburne University of Technology, Melbourne, VIC 3122, Australia; colin.hall@unisa.edu.au; 3 Future Industries Institute, University of South Australia, Adelaide, SA 5095, Australia; 4 Biomedical Manufacturing, CSIRO Manufacturing, Melbourne, VIC 3153, Australia

**Keywords:** mechanical properties, PECVD, plasma polymerization, thin films, TMDSO

## Abstract

Plasma-polymerised tetramethyldisiloxane (TMDSO) films are frequently applied as coatings for their abrasion resistance and barrier properties. By manipulating the deposition parameters, the chemical structure and thus mechanical properties of the films can also be controlled. These mechanical properties make them attractive as energy adsorbing layers for a range of applications, including carbon fibre composites. In this study, a new radio frequency (RF) plasma-enhanced chemical vapour deposition (PECVD) plasma reactor was designed with the capability to coat fibres with an energy adsorbing film. A key characterisation step for the system was establishing how the properties of the TMDSO films could be modified and compared with those deposited using a well-characterized microwave (MW) PECVD reactor. Film thickness and chemistry were determined with ellipsometry and X-ray photoelectron spectroscopy, respectively. The mechanical properties were investigated by nanoindentation and atomic force microscopy with peak-force quantitative nanomechanical mapping. The RF PECVD films had a greater range of Young’s modulus and hardness values than the MW PECVD films, with values as high as 56.4 GPa and 7.5 GPa, respectively. These results demonstrated the varied properties of TMDSO films that could in turn be deposited onto carbon fibres using a custom-built RF PECVD reactor.

## 1. Introduction

Protective coatings have been created using organosilane coatings, predominately for polymer substrates. Siloxane precursors, such as hexamethyldisiloxane (HMDSO) [1,2,3] and tetramethyldisiloxane (TMDSO) [4,5,6], have been used to safely deposit these organosiloxane coatings via plasma-enhanced chemical vapour deposition (PECVD) for many years. TMDSO films can be deposited with controllable mechanical properties by increasing the O_2_ concentrations in the monomer feed, which results in harder films and higher Young’s moduli because of the increase of Si-O-Si bonds within the film [4,5,7]. TMDSO films are used in automotive, aerospace, and biomedical applications to act as barrier coatings and to resist scratching and crazing [6]. A number of studies have investigated the mechanical properties of HMDSO films [8,9,10,11,12], but significantly fewer have investigated TMDSO films [4,5,7,13]. Harder TMDSO films with higher Young’s moduli have been deposited with higher O_2_ concentrations in the monomer feed because of the increase of Si-O-Si bonds within the film [4,5,7]. Very few studies have quantified the mechanical properties of microwave (MW) TMDSO films deposited by PECVD [4,5], with others performing semi-quantitative hardness analyses via pencil hardness testing [7,13]. Even fewer studies have investigated the deposition of TMDSO films deposited via radio frequency (RF) PECVD [14,15], let alone the mechanical properties. MW TMDSO films have been produced with hardness and Young’s modulus values of 0.014 to 1.2 GPa and 0.23 to 15.7 GPa, respectively.

Carbon fibres (CF) are frequently utilised to strengthen composite materials because of their high tensile strength, modulus, and wear resistance [16]. A critical component to consider in CF composites is the interface between the CF and the matrix, known as the ‘interphase’ [17], which transfers load between the two components and provides much of their mechanical performance [18]. Controlling the interphase via tailoring surface functionalities can manipulate the adhesion between the fibres and matrix and thus the overall performance of the composite [19,20]. Plasma polymer films have been deposited on various fibres to investigate the influence of specific surface functionalities on the interactions with the matrix, which hence influence the mechanical properties of the composite. Plasma polymer films have been shown to increase the tensile strength of fibres [21,22], and it has been suggested that these films fill in surface flaws present on the fibre in addition to acting as a protective layer [23]. Another advantage of plasma coatings was a reported increase in the stress transfer ability between fibres and matrix for plasma polymer films as thin as 5 nm [24]. Plasma polymer films are typically used for their chemical, rather than mechanical properties. Hence, the mechanical properties of these films are not commonly characterized. Previous research has however shown that organic ethyl lactate plasma polymer films had reduced modulus and hardness values of approximately 0.5 GPa and 9 GPa, respectively [25].

**Figure 1:**
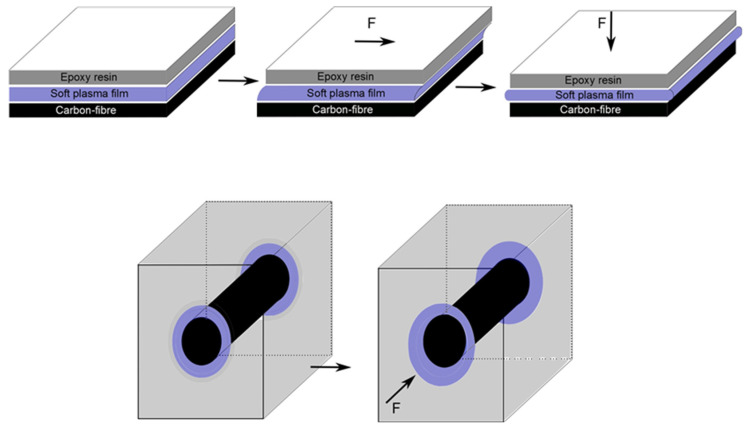
Schematic of a single layer plasma deposited film acting as a damping layer between fibres and matrix.

As illustrated in Figure 1, controlling the thickness and the mechanical properties of the interphase could positively influence the performance of CF composites [26]. It has been suggested that creating a composite interphase by inserting a plasma deposited layer between the fibre and the matrix will allow for a gradual change in stiffness while providing a strong bond [19]. The coating of fibres is, however, not straightforward, and a number of factors need to be considered to uniformly coat the fibres. Our previous work has shown that the position of the sample within a plasma polymerisation chamber relative to the electrode/s is a controlling factor in the chemistry and stability of plasma polymer films [27,28]. TMDSO films have been applied to CF composites to improve scratch resistance [13,29], but a continuous, or roll-to-roll, plasma deposition process for coating CFs prior to integration within a matrix to form a composite has yet to be demonstrated.

In a range of previous studies, MW PECVD TMDSO coatings have been produced via the addition of different oxygen concentrations into the monomer feed to produce films with a range of mechanical properties [4,5]. In this work, we compare the properties of these MW PECVD films to those produced at various powers using a dual-electrode RF PECVD reactor that was designed to enable future reel-to-reel fibre coatings [27,30]. The authors believe this is the first time that the mechanical properties of TMDSO films deposited by RF PECVD have been disseminated. Silicon wafer samples were positioned around a glass pipette to mimic the position CF, with subsequent analysis of both mechanical and chemical properties produced from a 100% TMDSO monomer deposited under different power regimes and electrode separations. The goal of the work is to aid in the understanding of how power and reactor design can be used to control film coating processes and the resulting film properties.

## 2. Results and Discussion

### 2.1. Thickness and Chemistry of TMDSO Films

To characterise the custom-built RF reactor for the deposition of TMDSO films, films were deposited at varying deposition powers to establish the range of mechanical properties that could be produced and then compared with the well-characterised MW TMDSO films. The MW TMDSO films were deposited with O_2_ concentrations in the monomer flow of 55–90%, whereas the RF TMDSO films were deposited without O_2_ to determine if the film properties were controllable in the absence of oxygen by varying only the deposition power. Our previous work optimising the RF power plasma reactor for radially uniform acrylic acid films demonstrated that the most uniform films resulted from a dual-electrode configuration [27]. Dual electrodes were therefore also utilized for the deposition of TMDSO films to optimise radial film thickness uniformity, under the assumption that this would also lead to greater uniformity in film chemistry and mechanical properties. The film thicknesses and X-ray photoelectron spectroscopy (XPS) atomic concentrations for RF TMDSO films deposited at three pairs of deposition powers are shown in Table 1. The difference in the powers for each electrode is based on the estimated power required to equalise the electron flux coming from each individual electrode, which was previously investigated using a Langmuir probe. For each deposition condition, the silicon wafer substrates were mounted axially around a glass pipette either parallel (0° facing E1, 180° facing E2) or perpendicular (±90°) to the electrodes. At an E1 power of 5 W, the 0° film had an average thickness of 236 nm. Thinner films were deposited at both ±90° and 180°, with the thinnest film of 204 nm resulting from the 180° sample. Such behaviour suggests that a power of 13 W for E2 is not sufficient to match the deposition conditions in front of E1. Thinner films were also previously observed for plasma polymerised acrylic acid films deposited perpendicular to the electrodes in this reactor [27], which was attributed to a shadowing effect that hinders energetic electrons from reaching the substrate [31]. At an E1 power of 20 W, the 0° film had an average thickness of 263 nm. As at 5 W, thinner films result from the samples perpendicular to the electrodes, with the 180° film again the thinnest. At 20 W, the films were typically approximately 25 nm thicker at each position in comparison with those at 5 W, with thicker films typically due to faster deposition rates at higher powers resulting from greater monomer fragmentation [32]. At a power of 50 W, with a decrease in the electrode spacing from between the electrodes from 160 mm to 80 mm, the 0° film had an average thickness of 127 nm, but unlike at 5 and 20 W, the ±90° and 180° films were thicker. At a sample position of 0°, the film thickness increased by approximately 30 nm, for an increase in the deposition power from 5 W to 20 W but a decrease by over 100 nm for a deposition power of 50 W. This reduction in film thickness, at the reduced electrode spacing of 80 mm, is less favourable when aiming for radially uniform film thicknesses. A competitive ablation and polymerisation process [33] occurs during plasma polymerisation. The films deposited at 50 W were thus thinner than those deposited at lower powers because of the switch from a deposition dominant regime to an ablation dominant regime, which occurs at both higher powers and closer to the electrode. The samples facing the electrodes (0° and 180°) are likely to be subject to greater ablation than the samples perpendicular to the electrodes (±90°), which accounts for their reduced film loss. Thinner films are also frequently observed at higher powers [32,34]. TMDSO film thickness was influenced by the electrode spacing as well as the deposition power, indicating that an electrode spacing of 160 mm produces the most radially uniform films, with careful power matching required when depositing a film using dual electrodes.

Analysis of XPS survey scans indicated that all RF TMDSO films were only composed of carbon, oxygen, and silicon, which was expected for the TMDSO monomer with a chemical structure of C_4_H_14_OSi_2._, as shown in Table 2. The chemistry of the samples deposited at 5 W had constant carbon, oxygen, and silicon concentrations of approximately 52%, 21%, and 27%, respectively, irrespective of sample position, which correlated with O/C and O/Si ratios of approximately 0.4 and 0.8, respectively. The surface chemistry of the samples deposited at 20 W showed variations resulting from the radial sample position. The 0° sample, which was the thickest film, had the lowest carbon content of 49.8% accompanied by the highest oxygen content of 23.4%. The ±90° and 180° samples, which were thinner than the 0° film, had slightly higher carbon concentrations of 50.7 to 50.9% and lower oxygen concentrations of 22.4 to 22.7%. The silicon content was not affected by the power mismatch and remained constant at approximately 27%. Films deposited at 50 W had inverse behaviour to the films deposited at 20 W, where the highest carbon and lowest oxygen concentrations were observed for the thinnest film at a sample position of 0°. Such behaviour suggests that the ablation dominant regime in front of E1 may produce more carbon-rich films. For all deposition powers, differences in the surface chemistry were most pronounced for the samples deposited at 0°, with relatively consistent silicon, carbon, and oxygen concentrations of approximately 27%, 50%, and 23%, respectively, for positions of ±90° and 180°. Such behaviour suggests that more uniform chemistries are likely to be produced by increasing the power of E2 to better match the conditions of E1. Overall, increasing the deposition power resulted in greater changes in the elemental composition for sample positions of ±90° and 180° than at 0°. For carbon, little overall change was observed at 0°, but a decrease was observed from approximately 52% to 49% for ±90° and 180°. The oxygen concentrations increased from 21% to 24%, while the silicon concentration stayed relatively constant at approximately 27%. The increases in power also increased both the O/C and O/Si ratios.

An MW reactor for the deposition of TMDSO films has been previously been well characterised [4,5,6]. In order to benchmark the RF reactor for the deposition of TMDSO films, MW TMDSO films were deposited under comparable conditions and the properties of the two types of TMDSO films were compared. The film thicknesses and XPS atomic concentrations for MW TMDSO films deposited at varying powers and O_2_ concentrations are shown in Table 3. In comparison with the RF TMDSO films, which had a maximum deposition rate of 13 nm/min, the MW reactor results in significantly faster deposition with average rates of 0.8–4.1 µm/min. As with RF TMDSO films, the MW TMDSO films are only composed of carbon, oxygen, and silicon. The chemistry of the MW TMDSO films was directly related to the O_2_ concentration during deposition. Higher oxygen concentrations in the MW TMDSO films result from higher O_2_ concentrations in the monomer feed, which are accompanied by lower carbon concentrations at both 1 and 2 kW. Both RF and MW TMDSO films had silicon concentrations similar to that of the initial TMDSO monomer at 28%. The deposition power, however, slightly influenced the oxygen and silicon concentrations, which were both higher at 2 kW than at 1 kW. The overall decrease in carbon and the increase in oxygen indicates oxygen incorporation into the Si-O-Si network, with previous studies linking higher O_2_ concentrations in the monomer flow to highly crosslinked Si-O-Si networks [6]. The MW TMDSO films, in which O_2_ was added to the monomer feed during the deposition, had higher oxygen concentrations than the RF TMDSO films which were deposited with TMDSO alone. MW TMDSO films had oxygen concentrations ranging from 36 to 57%, depending on the deposition power and O_2_ concentration in the monomer feed. The RF TMDSO films, however, had both lower and less variable oxygen concentrations of between 21% and 24%.

**Figure 2:**
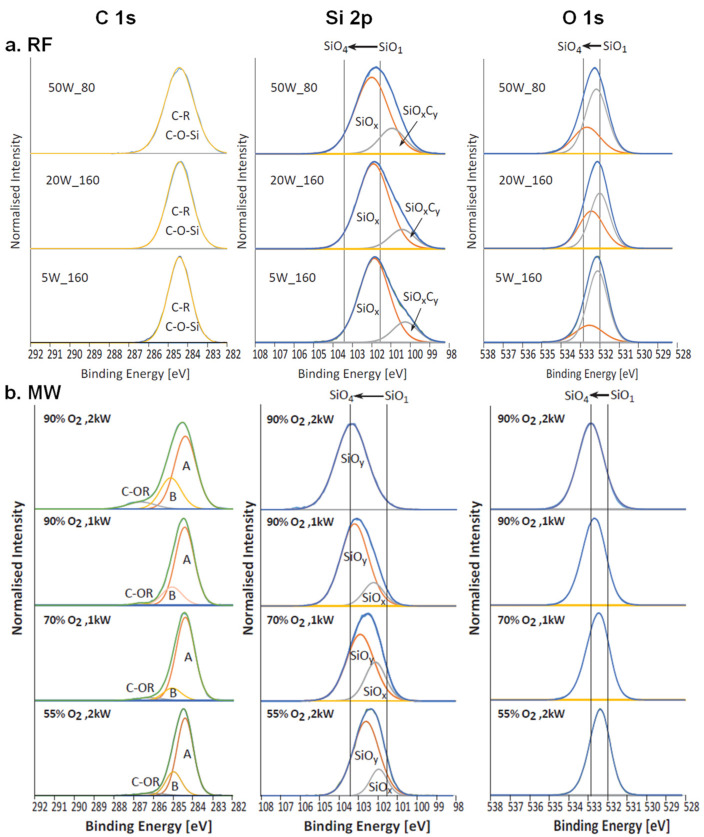
XPS high-resolution C 1s, Si 2p, and O 1s spectra for (**a**) RF and (**b**) MW TMDSO films deposited at varying powers and O_2_ concentrations.

High-resolution C 1s, O 1s, and Si 2p XPS spectra for RF and MW TMDSO films were analysed and are shown in Figure 2. The peak assignments for siloxane polymers were adapted to the TMDSO films produced in this study. For siloxane plasma polymers, such as TMDSO, the majority of the carbon is present as Si-CH_3_ and Si-CH_2_-CH_2_, with the C-C, C-H peak situated at 284.4 eV [35,36]. As plasma-deposited films are not conventional polymers, they have no repeating units, which makes curve fitting the XPS spectra to the extent present in the literature [37] challenging. During plasma polymerisation, depending on the power applied, the monomer can be severely fragmented, resulting in the chemistry of the deposited film having little resemblance to that of the starting monomer. Detailed curve fitting of the high-resolution TMDSO XPS spectra was not attempted because of the complex molecular structure of TMDSO plasma deposited films. A single symmetric Gaussian peak was fitted to the C 1s spectra, contributed to by C-R and C-O-Si components [36,37], shown in Figure 2a. The hydrocarbon peak was positioned to 284.4 eV and used for charge correction of the Si 2p and O 1s components. Increasing the deposition power resulted in a broadening of the C 1s peak, with an increase in the FWHM from 1.36 to 1.73, which may be attributed to the incorporation of additional oxygen into the TMDSO films, as shown in Table 2. The RF TMDSO Si 2p spectra were fitted with two components, which were assigned to SiO_x_ and SiO_x_C_y_. The positions of the SiO_x_ and SiO_x_C_y_ components were shifted towards higher binding energies as the deposition power was increased from 101.9 eV to 102.1 eV and 100.3 eV to 101 eV, respectively, which also resulted in a change in peak shape from a slightly asymmetric to a symmetric peak. The higher energy shift in the SiO_x_ component suggests an increase in oxygen atoms from SiO to SiO_2_ [37]. The O 1s spectra were also fitted with two components to help visualise the minimal shift of 0.2 eV to higher binding energies, which supports the slight oxygen incorporation observed as the deposition power was increased in the Si 2p spectra. The peak shape and FWHM of the individual components of the O 1s were not influenced by increasing deposition power. The slight peak shifts in the O 1s and Si 2p spectra at higher powers indicate the incorporation of additional oxygen into the RF deposited TMDSO film, which correlates with the elemental composition of oxygen increasing from 21% to 24%. The reduction in carbon and increase in oxygen concentrations as the deposition power was increased is attributed to the TMDSO fragmentation mechanism. Higher powers likely result in greater fragmentation of the TMDSO molecule, which cleaves the methyl groups of the TMDSO molecule, which again results in a more ‘silica like’ film with a crosslinked network of O-Si-O [4].

The curve-fitted high-resolution C 1s, Si 2p, and O 1s spectra of MW TMDSO films deposited at varying powers and O_2_ concentrations are shown in Figure 2b. In comparison to the RF TMDSO films, the higher oxygen concentrations in the MW TMDSO films produced C 1s spectra that enabled the fitting of three peaks to the data. Peak A represents the contributions from Si-C, C-C, and Si-H, with the Si-C peak situated at a lower energy than the C-C, C-H peak at approximately 283 eV [38]. Peak B, at approximately 285 eV, was assigned to the beta-shift of the hydrocarbon peak, possibly because of a chain structure such as C-C-O-Si-O in which the oxygen atoms cause a shift to higher binding energies. The smaller peak at approximately 286–286.5 eV was assigned to C-OR bonds. At 90% O_2_, the C 1s peaks broaden towards higher binding energies because of the increased contribution of the C-OR component. The binding energies of siloxane groups range from 101.6 to 103.5 eV for Si 2p and 532.0 to 532.8 eV for O 1s, with higher binding energies for the MW than the RF spectra, which suggests the replacement of C-H_x_ groups by oxygen atoms [36,37]. The XPS high-resolution O 1s and Si 2p spectra, in combination with the elemental composition, indicate the greater incorporation of oxygen into highly crosslinked Si-O-Si networks. Increasing the O_2_ concentration in the monomer flow and deposition power produced peak shifts towards higher binding energies, indicating a transition from Si-O_2_ to Si-O_4_ networks. The high-resolution XPS spectra showed that RF TMDSO films had a much greater contribution of SiO_x_C_y_ in the Si 2p spectra than the MW TMDSO films, which is attributed to the lower oxygen and higher carbon concentrations. The high-resolution O 1s spectra for both MW and RF TMDSO films showed shifts to higher binding energies, with increasing deposition power and/or O_2_ concentration in the monomer feed for MW TMDSO. Such behaviour is attributed to additional oxygen incorporation into the film for MW TMDSO and greater monomer fragmentation for both film types, resulting in loss of methyl groups and leading to a greater contribution of the Si-O-Si backbone. The shifts for the MW TMDSO films due to their higher oxygen concentrations indicated a transition from SiO_2_ towards SiO_4_, while for RF TMDSO films, these shifts indicated a transition from SiO to SiO_2_.

### 2.2. Mechanical Properties of TMDSO Films

The influence of the deposition power on the mechanical properties of RF and MW TMDSO films was investigated with nanoindentation and atomic force microscopy using peak-force quantitative nanomechanical mapping (AFM PF-QNM). Nanoindentation showed increases in both Young’s moduli, which indicates the stiffness of the film, and the hardness from approximately 2 GPa to 56 GPa and 0.2 GPa to 7.5 GPa, respectively, for RF TMDSO films, with an increase in deposition power from 5 W to 50 W, as shown in Table 4. A deposition power of 5 W appeared to test the lower limits of the nanoindenter because of its low crosslinking density in combination with a thickness of around 200 nm. At the lowest load that still produced a reasonable force-displacement curve (2 μN), the nanoindenter measured a modulus of 2.1 GPa for the 0° film, but kept increasing to 3.5 GPa for a load of 3 μN and further to 4.7 GPa at a load of 5 μN. These increases in modulus with load indicate a substrate effect that thus brought the nanoindentation results for films deposited at 5 W into question. Films deposited at 20 W and 50 W for a sample position of 0° had Young’s modulus values around 6 GPa and 56 GPa, respectively, up to a normalised indentation depth up of 0.3. The average Young’s modulus and hardness values had standard deviations of 8 to 14%, indicating relatively consistent results for a normalised indentation depth of up to 0.3. For deposition powers of 5 W and 20 W, both with an electrode spacing of 160 mm, the mechanical properties of the films were similar, irrespective of being positioned facing (0°) or perpendicular (90°) to the electrode. For the closer electrode spacing of 80 mm at 50 W, there were significant differences in the Young’s modulus and hardness between the 0° and 90° samples. The film facing the electrode had approximately 60% higher Young’s modulus and hardness values in comparison with the film deposited perpendicular to the electrode. Such behaviour demonstrates that films deposited when facing the electrode were stiffer and harder, which was attributed to greater fragmentation of the TMDSO monomer resulting in a more crosslinked film. The TMDSO film deposited at 50 W and 0° had Young’s modulus and hardness values in a similar range to fused silica/glass [5,39], thus providing the context of the mechanical properties that can be produced via plasma deposition. Both the range of mechanical properties and the maximum hardness or Young’s modulus were also greater than those previously reported for inorganic HMDSO films deposited via atmospheric [40], direct current [41], or RF plasma [42].

AFM PF-QNM was also used to investigate the stiffness and the roughness of RF TMDSO films, and it appeared to be a more suitable technique to analyse the softer films deposited at lower power. The roughness of all of the RF films was less than 3 nm, with the films deposited at 50 W slightly smoother than those at lower powers. Sample position did not influence surface roughness, with similar R_q_ values at both 0° and 90°, even for those at 50 W, which had significantly different Young’s moduli. The Derjaguin, Muller, Toropov (DMT) modulus values were also relatively consistent around the circumference of the glass rod when taking into account that the standard deviation values made a distinction between the films deposited at 0° and 90° unreliable. When the deposition power was increased from 5 W to 20 W, the average DMT-modulus of the TMDSO films deposited at 0° increased from 2.8 GPa to 10.1 GPa, with a further increase to 26.2 GPa at 50 W. These increases coincide with the nanoindentation Young’s modulus values, demonstrating that higher deposition powers produce stiffer films because of increased fragmentation of the TMDSO monomer. The large standard deviations for the DMT-modulus values of the TMDSO films deposited at 50 W suggest that the reliable limit of the AFM Tap525 probe may be around 20 GPa because of the highly variable results produced for DMT-modulus values of ≥20 GPa. Varying the deposition power successfully controlled the mechanical properties of TMDSO films. A range of Young’s moduli from 2 to 56 GPa was achieved for deposition powers of 5–50 W, respectively, which were attributed to the transition from a more organic-rich to a more crosslinked inorganic O-Si-O structure.

**Figure 3:**
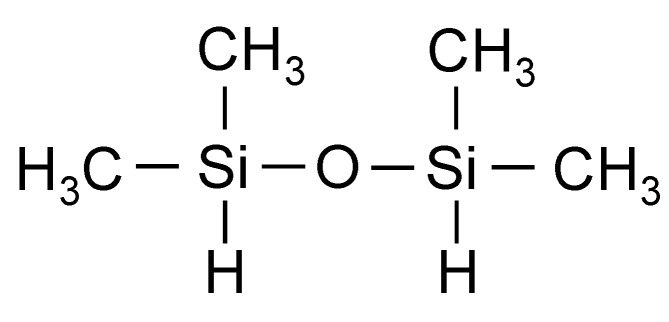
Structure of TMDSO.

The mechanical properties of the MW TMDSO films were also measured using nanoindentation and AFM PF-QNM and are shown in Table 4. The Young’s modulus and hardness values ranged from 1.3 GPa to 10.9 GPa and 0.25 GPa to 1.05 GPa, similar to those deposited previously for MW TMDSO films [4,5]. Significantly higher Young’s modulus and hardness values were, however, achieved by the RF TMDSO films, with values as high as 56.4 GPa and 7.5 GPa, respectively, without the addition of O_2_. For MW TMDSO films, stiffer films resulted from a higher O_2_ concentration, which has previously been observed for TMDSO films deposited in this reactor [4,6]. As for the RF TMDSO films, deposition power, however, also influences the Young’s modulus for MW films, with higher values at 2 kW than 1 kW for an O_2_ concentration of 90%. The higher concentration of O_2_ within the system results in greater fragmentation of the TMDSO molecule, which likely results in the efficient cleavage of the methyl groups, as shown in the TMDSO structure in Figure 3, leaving a relatively bare O-Si-O backbone. Such fragmentation results in a stiffer, more ‘silica-like’ film with a crosslinked network of O-Si-O [4]. The high-resolution Si 2p and O 1s spectra demonstrate the shift from Si-O_2_ to Si-O_4_, indicating the formation of highly crosslinked Si-O-Si networks at higher O_2_ concentrations in the monomer feed. The increases in Young’s modulus values for both RF and MW TMDSO films are attributed to greater monomer fragmentation, leading to greater crosslinking, which results from higher deposition powers and/or O_2_ concentrations in the monomer feed. The RF PECVD reactor, however, despite having no O_2_ in the monomer feed, produced TMDSO films with higher Young’s modulus values than the MW TMDSO PECVD reactor. Such behaviour indicates that the RF power alone, without O_2_ enhanced monomer fragmentation, is sufficient to induce crosslinking in TMDSO films. Such crosslinking at higher powers is frequently observed for plasma polymer films [32,43,44].

The majority of the MW TMDSO films were slightly smoother than the RF TMDSO films, with R_q_ values as low as 0.4 nm. The film deposited with 90% O_2_ at 2 kW, which had an average R_q_ roughness of 9.6 nm, suggests that at a higher O_2_ concentration, increasing the deposition power influences surface roughness. The ISO14577 standard for nanoindentation states that the surface roughness should be less than 5% of the maximum penetration depth. This is not an issue for the R_q_ roughness values of 1–2 nm. On the basis of this stipulation, the surface roughness of the TMDSO film deposited with 90% O_2_ at 2 kW of 9.6 nm indicates that the maximum penetration depth should be 192 nm, which is less than the maximum indentation depth of approximately 120 nm (normalised indentation depth of 0.3) used to calculate the average Young’s modulus. The MW TMDSO films have DMT-modulus values up to 8.6 GPa, with higher values for higher O_2_ concentrations. Higher DMT modulus values, as with the Young’s Modulus, could be produced by RF TMDSO films. Higher standard deviations for both the Young’s modulus determined via nanoindentation and the DMT modulus determined via PF-QNM for the 90% O_2_ at 2 kW MW TMDSO films were attributed to the surface roughness. A standard deviation close to 50% suggests that such surface roughness may be a limiting factor for PF-QNM.

Comparing the Young’s moduli determined by nanoindentation and the DMT modulus or the reduced Young’s modulus determined by PF-QNM showed that for TMDSO films deposited at the lower powers and/or O_2_ concentrations, the Young’s and DMT moduli did not differ significantly. Although nanoindentation and AFM PF-QNM both successfully measured the mechanical properties of TMDSO films over a wide range of elastic moduli, the differences in the Young’s and DMT moduli highlight their suitability as film stiffness changes. Nanoindentation with a sharp cube corner indenter increased the sensitivity of this technique significantly, but it still reached its limits for thin (<1 μm) and soft films (<1 GPa), which resulted in the observation of an indirect substrate effect. The softest films for RF TMDSO were less accurately analysed via nanoindentation than the softest MW TMDSO films, which actually had lower Young’s Modulus values because of their low crosslinking and because they were thinner films (~400 nm vs. 200 nm), which resulted in substrate effects. These results suggest that nanoindentation requires a certain film thickness for soft films to avoid the indirect substrate effect. As AFM PF-QNM appears to be less affected by the combination of soft and thin films, it produces more reliable stiffness measurements for softer films, with values around 2 GPa, and is thus better suited for the low modulus range than nanoindentation because of its greater sensitivity. AFM PF-QNM, however, reached its limitations for stiff (>20 GPa) and rough (>5 nm) films, with nanoindentation producing values with lower standard deviations for these films. The combination of both techniques appears ideal to provide reliable results for TMDSO films deposited with a wide range of mechanical properties. These differences in the nanoindentation and AFM PF-QNM techniques can be highlighted by comparing the RF TMDSO deposited at 20 W and 50 W and the MW TMDSO film deposited at 2 kW with 90% O_2_. For the 20 W RF film, in which both nanoindentation and AFM PF-QNM are likely to produce reliable stiffness results, a lower Young’s modulus than DMT modulus was observed, but the difference was small when the standard deviations are taken into account. However, for the significantly stiffer RF film deposited at 50 W, nanoindentation results in a significantly lower standard deviation than for AFM PF-QNM. For the MW film, AFM PF-QNM produces a noticeably lower DMT modulus than the Young’s modulus determined via nanoindentation, which was expected to be related to its higher roughness. The low load used for PF-QNM results in penetration depths of around 2–3 nm, which were well below the surface roughness of the film. It was reported that surface roughness significantly reduced the measured modulus and hardness values for indentation depths below the surface roughness [45]. The higher load range of nanoindentation and the deeper indentation depth appeared to reduce the influence of the surface roughness on the modulus measurements. The nanoindentation Young’s modulus of 10.9 GPa was therefore considered more realistic than the AFM PF-QNM result of 5.9 GPa and was in agreement with the values reported in the literature [4].

PECVD TMDSO films deposited using MW and RF powered chambers appear to follow different pathways to achieve improved mechanical properties. The MW PECVD process uses O_2_ in the monomer flow to create oxygen radicals, which fragments the TMDSO monomer and provides oxygen in surplus to promote Si-O-Si bond formation. Hence, the increase in O_2_ content in the monomer flow may result in increased monomer fragmentation and provide more oxygen atoms in the system to form a ‘silica glass-like’ structure. This was in agreement with the XPS analysis, which showed a 21% increase in oxygen concentrations and a transition from Si-O_2_ to Si-O_4_ structures. In the RF PECVD process, in which no O_2_ was added to the monomer feed, fragmentation and recombination of the monomer were controlled by the deposition power, which led to higher monomer fragmentation and thus crosslinking at higher powers, with only a 2% increase in the oxygen concentrations within the films. Previous analysis of TMDSO has shown that deposition at higher powers for RF reactors [14] and at higher O_2_ concentrations in the monomer feed for both RF and MW reactors [6,14] reduces the concentration of methyl groups and increases the Si-O groups within the film, producing a more ‘silica-like’ structure. These results support the supposition that the increased mechanical properties of both the MW and RF deposited TMDSO films result from greater crosslinking, albeit via different mechanisms. The attenuated total reflection Fourier transform infrared (ATR-FTIR) spectra of the RF TMDSO films (Figure S1) show a broadening of the Si-O-Si peak and a decrease in the Si-H and Si-C peaks at higher powers. These results suggest greater crosslinking in the films at higher powers and the transition from a silicone to a more ‘silica-like’ film, both of which account for the increased mechanical properties at higher powers.

## 3. Materials and Methods

### 3.1. Materials

TMDSO (97% purity) was purchased from Sigma Aldrich (Sydney, Australia), with its structure shown in Figure 3. Decon 90 was purchased from Bio-Strategy Pty. Ltd. (Melbourne, Australia). The <100> orientation, single side polished, thickness 600–650 µm silicon wafer substrates were purchased from Micro Materials and Research Consumables Pty. Ltd. (Melbourne, Australia).

### 3.2. Substrate Preparation

Silicon wafer substrates were cleaned prior to TMDSO deposition by sonicating in 5% Decon 90 solution for 30 min, rinsed with Milli-Q water (18.2 MΩ, Millipore, North Ryde, Australia), and dried with compressed air.

### 3.3. MW TMDSO Films

Oxygen pre-treatment and the deposition of TMDSO films were performed using a custom-built MW PECVD system, which was previously described [5]. Briefly, two copper antennas were semi-enclosed in the centre of four aluminium oxide tubes and located along one wall inside the chamber to form the plasma zone. A power of 1–2 kW at a frequency of 2.45 GHz was introduced at both ends of the copper antennas to create an evenly distributed plasma field. O_2_ was introduced into the chamber behind the copper antennas while TMDSO vapour was introduced separately through a showerhead design in close proximity to the substrate with the monomer outlets facing the substrate. The monomer and O_2_ flows were adjusted by Low∆P (Bronkhorst, Ruurlo, The Netherlands) and UFC-8100 (Unit Instruments, Hatfield, PA, USA) mass flow controllers. The vacuum system was comprised of an E2M80 rotary pump (Edwards, UK) in combination with an EH500 Roots blower pump (Edwards, UK), which were connected to pumping ports at the top and bottom of the chamber and monitored with a capacitance manometer (MKS Instruments, USA). The entire system was controlled by a programmable logic controller.

Prior to TMDSO deposition, the silicon wafer substrates were pre-treated with an oxygen plasma at 1 kW for 20 s with an O_2_ flow rate of 400 standard cubic centimetres per minute (sccm). The TMDSO films were deposited from a mixed vapour phase comprised of the TMDSO monomer and ultrahigh purity O_2_ gas. Previously obtained deposition conditions [4] were used to deposit films with mechanical properties ranging from soft to hard by increasing the O_2_ concentration from 55% to 90%. The combined flow rate of TMDSO and O_2_ was kept constant at approximately 444 sccm. The O_2_ concentrations in the monomer feed were set at 70% and 90% for 1 kW and 55% and 90% for 2 kW, which corresponded to TMDSO/O_2_ flow rates of 133/311, 44/400, 199/245, and 44/400 sccm, respectively. The observed pressure during deposition was ≈0.46–0.52 mbar. The deposition time for each deposition condition was adjusted by performing multiple runs to achieve thicknesses of approximately 400 nm. All samples were stored for 1 month in ambient conditions prior to testing to ensure that all possible aging effects of the TMDSO films had already occurred [4].

### 3.4. RF TMDSO Films

**Figure 4:**
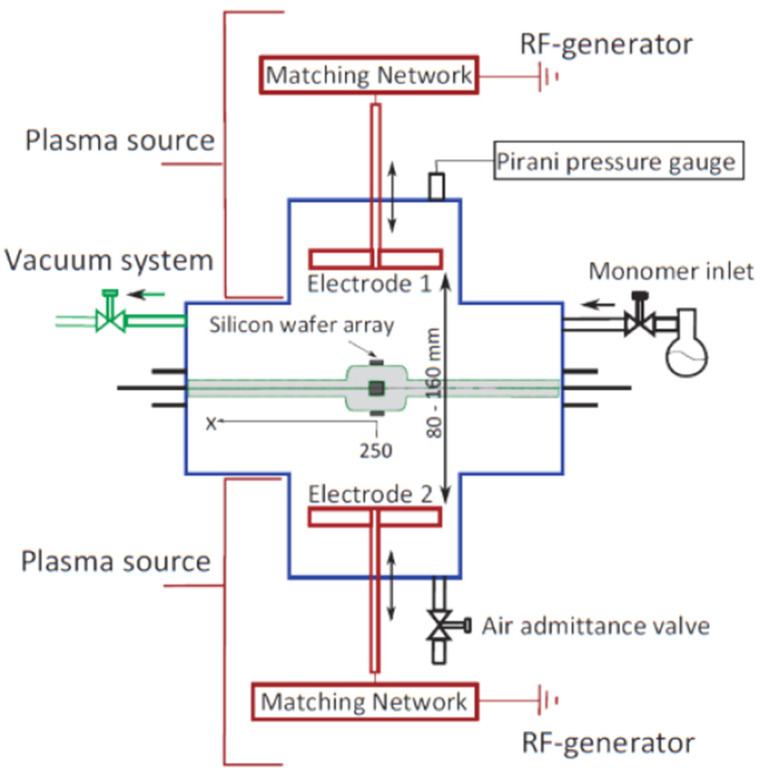
Schematic top view diagram of the RF PECVD chamber.

TMDSO films were also deposited using an RF PECVD reactor, which was previously described [27]. A schematic diagram of the chamber is shown in Figure 4. Briefly, the reactor was comprised of two electrodes (E1 and E2) within a stainless-steel ISO-200 4-way cross with four ISO-200 flanges sealed with centring rings and Viton O-rings. The plasma was ignited by a 13.56 MHz RF generator (RFG100-13, Coaxial Power Systems Ltd., Eastbourne, UK), and a manual matching network (MMN150; Coaxial Power Systems Ltd., Eastbourne, UK) to minimize reflected power was connected to each electrode. The vacuum system was comprised of a cold trap, two z-line valves, and a rotary vane pump (RV 12; Edwards Ltd., UK). The pressure within the chamber was measured using a Pirani pressure gauge (APG100XLC-KF16, Edwards Ltd., Burgess Hill, UK). The reactor was also built with two internal removable spools to enable the future coating of CFs, which would run through the centre of the chamber from one spool to the other, 80 mm from each electrode. The spool-to-spool transfer and stepper motor control unit would be attached to a KF-50 flange on each side of the reactor. This spool-to-spool attachment was removed during this study and replaced with a glass pipette to mimic the position of the CF to characterize TMDSO deposition in this chamber.

The liquid TMDSO was placed in a round bottom flask and the vapour was admitted to the chamber using a needle valve (CMV-VFM-3-P-KK, Chell Instruments Ltd., North Walsham, UK). Each deposition required filling the cold trap with liquid nitrogen and evacuating the plasma chamber to a base pressure of approximately 1 × 10^−3^ mbar followed by a leak test. The TMDSO monomer was degassed prior to deposition using three freeze-thaw cycles. The TMDSO films were deposited with a distance between the electrodes of either 80 or 160 mm at deposition powers of 5, 20, or 50 W for the front electrode (E1) and a flow rate of 2 sccm for 20 min. The 1 cm^2^ silicon wafer substrates were mounted at four positions around a glass pipette with double-sided tape, either parallel (0° or 180°) or perpendicular (±90°) to the electrode, which was positioned down the centre of the chamber to mimic the future position of CF.

### 3.5. Spectroscopic Ellipsometry

The thickness of the TMDSO films was measured in triplicate using an M-2000XI Ellipsometer (J. A. Woollam Co. Inc., Lincoln, NE, USA) at a wavelength range of 210.9–1687.7 nm and at angles of 45° to 65° in 5° increments. The ellipsometry data were processed with CompleteEASE software version 4.90 (J. A. Woollam Co. Inc., Lincoln, NE, USA). The acquired data were fit with a model which consisted of a silicon substrate, 1 nm native SiO_2_, and a B-spline layer for the TMDSO film. The model was fit in Kramers–Kronig mode with force E^2^ positive set within the advanced settings.

### 3.6. XPS

The chemistry of the TMDSO films was analysed by XPS with an AXIS Nova (Kratos Analytical Inc., Manchester, UK) with a monochromated Al Kα X-ray source (λ = 1486.6 eV). The X-ray spot size was a slot with dimensions of 700 μm × 300 μm. The pressure within the analysis chamber during analysis was typically below 10^−8^ mbar. The photoemission angle of 0° corresponded to a 90° take-off angle respective to the surface. The survey and high-resolution C 1s spectra were collected at pass energies of 160 eV and 20 eV, respectively, and scan times of two minutes with two sweeps each. Three spots were analysed per sample with the electron gun used for charge neutralization. The acquired spectra were analysed using Casa XPS software version 2.3.15 (Casa Software Ltd., Cheshire, UK), with element quantification based on the standard relative sensitivity factors provided by the manufacturer. The spectra were calibrated using the hydrocarbon (C-C) to 285 eV.

### 3.7. AFM

Mechanical properties and surface topography of the TMDSO films were assessed by AFM PF-QNM. PF-QNM images surface topography similarly to conventional AFM in tapping mode but simultaneously records the nanomechanical properties of the sample. A wide modulus range of 1 MPa to 50 GPa can be measured in conjunction with the correct probe. A Bruker MultiMode 8 AFM in combination with Tap300 (55/75% O_2_ for MW, 5 W for RF) and Tap525 (90% O_2_ for MW, 20/50 W for RF) probes was used to determine the reduced Young’s modulus of TMDSO films on silicon wafer substrates. The average DMT-modulus (reduced Young’s modulus) and roughness values were extracted from three scans per sample, which translated to over 500,000 individual peak force/height measurements for each sample. The surface topography and DMT-modulus scans are shown in Figure S2. Calibration of the probe parameters (deflection sensitivity, cantilever stiffness, and tip radius) was required to ensure accurate results. The AFM PF-QNM had a force range of 10 pN to 10 μN, and the applied force was precisely controlled to prevent sample damage. The sampling depth was typically limited to a few nanometres, and ScanAsyst was used to ensure optimised sampling conditions. During imaging, force curves were recorded for each point. These force curves were then converted into force-separation curves, which are analogues to the load-displacement curves recorded during nanoindentation experiments. PF-QNM extracts four material parameters from the force-separation curves: reduced Young’s modulus, adhesion, dissipation, and deformation. The reduced Young’s modulus was obtained by fitting the retract curve using the Derjaguin, Muller, Toropov (DMT) model [46]. The surface topography and the mechanical properties were analysed by the Nano_scope software using the ‘Height’ channel and the ‘DMT modulus’ channel, respectively. The average surface roughness (R_q_) was obtained by flattening the height image and using the roughness option. The average DMT-modulus for each scan was extracted using the ‘Image Raw Mean’ of the ‘DMT’ channel, while the standard deviation correlated with the ‘Image R_q_’ and was averaged for the three scans using the square root of the variance. This was performed for three scans with a scan size of 3 µm. PF-QNM measurements have an accepted error of ≈25% since the Poisson’s ratio of the sample is typically unknown and can range from 0.2–0.5 [47]. In this study, a Poisson’s ratio of 0.3 was assumed for all samples and allowed direct comparison of the DMT-modulus and the Young’s modulus obtained from nano-indentation. To ensure the validity of the measured results, a reference sample was used after each tip calibration and between measurements. The reference sample was comprised of distinct regions of polystyrene (PS) and PS/low-density polyethene copolymer with known elastic moduli of ≈2 GPa and ≈0.1 GPa, respectively.

### 3.8. Nano-Indentation

Nano-indentation was performed using a Hysitron Premier Ti (Hysitron Inc., Minneapolis, MN, USA), which had a load range of 70 nN to 10 mN and measured displacements from 2 Å to 5 μm. A sharp cube corner tip was used to perform the indentation experiments as suggested in the literature [48]. Nanoindentation creates a permanent indent, which allowed the calculation of the hardness and stiffness (Young’s modulus) of the material. It was assumed that the cube corner in combination with the Hysitron’s load range and high displacement accuracy had the potential to measure mechanical thin film properties at shallow depths. The onset of plastic deformation was determined by repeated load–unload cycles and steadily increasing maximum load to the point at which a residual indent remained in the film. Surface topography scans were performed after each applied load to verify the onset of plastic deformation in the form of a permanent indent. The Hysitron TriboView software was used to analyse the surface topography scans in regard to surface roughness and plastic deformation. The load-displacement curves recorded with the Hysitron nanoindenter were analysed using the Oliver–Pharr method, which requires measurements to be performed in the plastic regime to ensure the applicability of the method [49,50]. Three indents were performed for each applied load to analyse the onset of substrate effect, and all samples were indented to a minimum of 50% of their film thickness. The hardness of the film was calculated by dividing the maximum load, obtained from the load-displacement curves, by the contact area. The Young’s modulus (*E*) was obtained using the following equation:

(1) $$\frac{1}{E^{*}}=\frac{1-v^{2}}{E}+\frac{1-v_{i}^{2}}{E_{i}}$$

where E* is the nanoindentation reduced Young’s modulus, *v* is the Poisson’s ratio of the material (0.3), *v_i_* is the Poisson’s ratio of the indenter (0.07), and *E_i_* is the Young’s modulus of the indenter (1140 GPa)

Surface topography scans were performed after each applied load to verify a permanent indent in the film (Figure S3). The surface topography images recorded after the indentation experiment determines the depth of the plastic deformation after the films have elastically recovered, while the nanoindenter records the actual penetration depth during the indentation experiment. For nanoindentation, the substrate influences the results once a certain indentation depth is reached, and then the mechanical properties increase with increasing indentation depth [48,51]. Young’s modulus measurements up to a normalised penetration depth of 0.3 (100–150 nm) were possible before increases in the moduli were visible, indicating the onset of substrate effect (Figure S4), which is deeper than has been previously reported in the literature [51,52,53]. The Young’s modulus and the hardness values results were thus averaged for all indentations up to a normalised indentation depth of 0.3.

## 4. Conclusions

The range of mechanical properties that can be achieved for TMDSO films via a custom-built RF PECVD reactor was demonstrated. The authors believe this is the first time that the outcomes of such characterisation have been reported. In comparison with TMDSO films deposited in a well-characterised MW PECVD reactor, the RF films had significantly lower deposition rates and oxygen concentrations. The RF reactor, however, could deposit TMDSO films with higher Young’s modulus and hardness values than the MW reactor. The highest Young’s modulus and hardness values for MW TMDSO films were 10 GPa and 1.1 GPa, respectively, but were as high as 56 GPa and 7.5 GPa, respectively, for the RF TMDSO films. While harder and stiffer films were produced in the MW reactor by increasing the O_2_ concentration in the monomer feed, they could be achieved in the RF reactor just by increasing the deposition power for the RF reactor. A novel RF PECVD reactor with a double electrode configuration was built and successfully characterised for the deposition of TMDSO films, which were relatively radially uniform and had a range of mechanical properties that were controllable by varying only the deposition power.

## Figures and Tables

**Table 1:** Deposition parameters and ellipsometry film thicknesses for RF TMDSO films.

**Power (W)—E1**	5	20	50
**Power (W)—E2**	13	30	60
**Elecrode spacing (mm)**	160	160	80
**Sample Position**	**Film thickness (nm)**		
**0** **°** **(Facing E1)**	236 ± 2	253 ± 1	127 ± 6
**90** **°** **(Facing up)**	213 ± 9	244 ± 2	171 ± 1
**−90** **°** **(Facing down)**	221 ± 6	237 ± 2	177 ± 1
**180** **°** **(Facing E2)**	204 ± 1	228 ± 1	141 ± 1

**Table 2:** XPS atomic concentrations and O/C and O/Si ratios for RF TMDSO films deposited at various powers.

E1	Sample	XPS Atomic Concentration (%)				
Power (W)	Position	C	O	Si	O/C	O/Si
5	0°	51.9 ± 0.2	21.3 ± 0.2	26.8 ± 0.1	0.41	0.79
	90°	52.1 ± 0.1	21.2 ± 0.1	26.6 ± 0.1	0.41	0.80
	−90°	52.1 ± 0.3	21.2 ± 0.1	26.6 ± 0.2	0.41	0.80
	180°	52.2 ± 0.1	21.0 ± 0.1	26.8 ± 0.1	0.4	0.78
20	0°	49.8 ± 0.2	23.4 ± 0.1	26.8 ± 0.3	0.47	0.87
	90°	50.8 ± 0.2	22.6 ± 0.2	26.6 ± 0.1	0.45	0.85
	−90°	50.7 ± 0.1	22.7 ± 0.1	26.6 ± 0.1	0.45	0.85
	180°	50.9 ± 0.4	22.4 ± 0.1	26.7 ± 0.3	0.44	0.84
50	0°	51.9 ± 0.2	22.5 ± 0.1	25.7 ± 0.2	0.43	0.87
	90°	49.4 ± 0.6	23.7 ± 0.3	26.8 ± 0.2	0.48	0.88
	−90°	49.0 ± 0.2	24.1 ± 0.4	27.0 ± 0.2	0.49	0.89
	180°	49.7 ± 0.2	23.8 ± 0.3	26.5 ± 0.1	0.48	0.90

**Table 3:** Film thicknesses and XPS atomic concentrations of MW TMDSO films deposited at varying powers and O_2_ concentrations.

Power	O_2_	Deposition	Film Thickness	XPS Atomic Concentration (%)		
(kW)	(%)	Time (S)	(nm)	C	O	Si
1	70	6	414 ± 2	33 ± 1.0	42 ± 0.1	25 ± 0.1
1	90	30	529 ± 7	22 ± 0.6	51 ± 0.6	27 ± 1.0
2	55	25	343 ± 6	39 ± 0.1	36 ± 0.1	25 ± 0.1
2	90	20	393 ± 5	16 ± 0.4	57 ± 0.3	27 ± 0.2

**Table 4:** Mechanical properties for RF and MW TMDSO films obtained via cube corner nanoindentation and AFM PF-QNM at varying powers and O_2_ concentrations.

RF	Sample	MW Power	O_2_	Young’s Modulus	Hardness	R_q_-AFM	DMT-Modulus
Power (W)	Position	(kW)	(%)	(GPa)	(GPa)	(nm)	(GPa)
5	0°	-	-	2.1	0.2	2.1 ± 0.1	2.8 ± 0.8
	90°	-	-	2.2	0.2	2.2 ± 0.2	2.1 ± 0.5
20	0°	-	-	5.6 ± 0.7	0.96 ± 0.09	2.5 ± 0.6	10.1 ± 3.0
	90°	-	-	4.2 ± 0.6	0.73 ± 0.1	2.6 ± 0.1	7.0 ± 2.3
50	0°	-	-	56.4 ± 1.01	7.46 ± 1.01	1.6 ± 0.2	26.2 ± 7.4
	90°	-	-	34.7 ± 4.8	4.77 ± 0.8	1.4 ± 0.2	40.3 ± 18.3
-	-	1	70	1.31 ± 0.08	0.25 ± 0.03	0.4 ± 0.1	0.75 ± 0.04
-	-	1	90	6.38 ± 0.52	1.05 ± 0.13	0.7 ± 0.3	8.6 ± 0.9
-	-	2	55	1.31 ± 0.18	0.26 ± 0.05	0.6 ± 0.1	0.28 ± 0.01
-	-	2	90	10.87 ± 2.13	0.93 ± 0.24	9.6 ± 1.7	5.9 ± 2.6

## Data Availability

The data presented in this study are available on request from the corresponding author.

## Supplementary Materials

The following are available online. Figure S1: ATR-FTIR spectra of TMDSO and RF TMDSO films at various deposition powers. Spectra were offset along the y-axis for clarity. (Nicolet^TM^ iS^TM^ 5N FT-NIR spectrometer, 3 spots, 36 scans at a resolution of 8 cm^−1^), Figure S2: AFM surface topography scans and DMT-modulus maps of MW TMDSO films deposited at varying powers and O_2_ concentrations, Figure S3: Nanoindentation surface topography scans of MW TMDSO films deposited at varying powers and O_2_ concentrations after individual indents with increasing load. Scan sizes ranged from 0.5–2 µm depending on load conditions and indenter shape, Figure S4: Young’s modulus of MW TMDSO films measured by nanoindentation for varying O_2_ concentrations and deposition powers.
