# Supplementary figures and images for: Characterising a Custom-Built Radio Frequency PECVD Reactor to Vary the Mechanical Properties of TMDSO Films
Source: Molecules. 2021 Sep 16;26(18):5621. doi: 10.3390/molecules26185621 (PMC8468313; doi:10.3390/molecules26185621)

## Slide 1
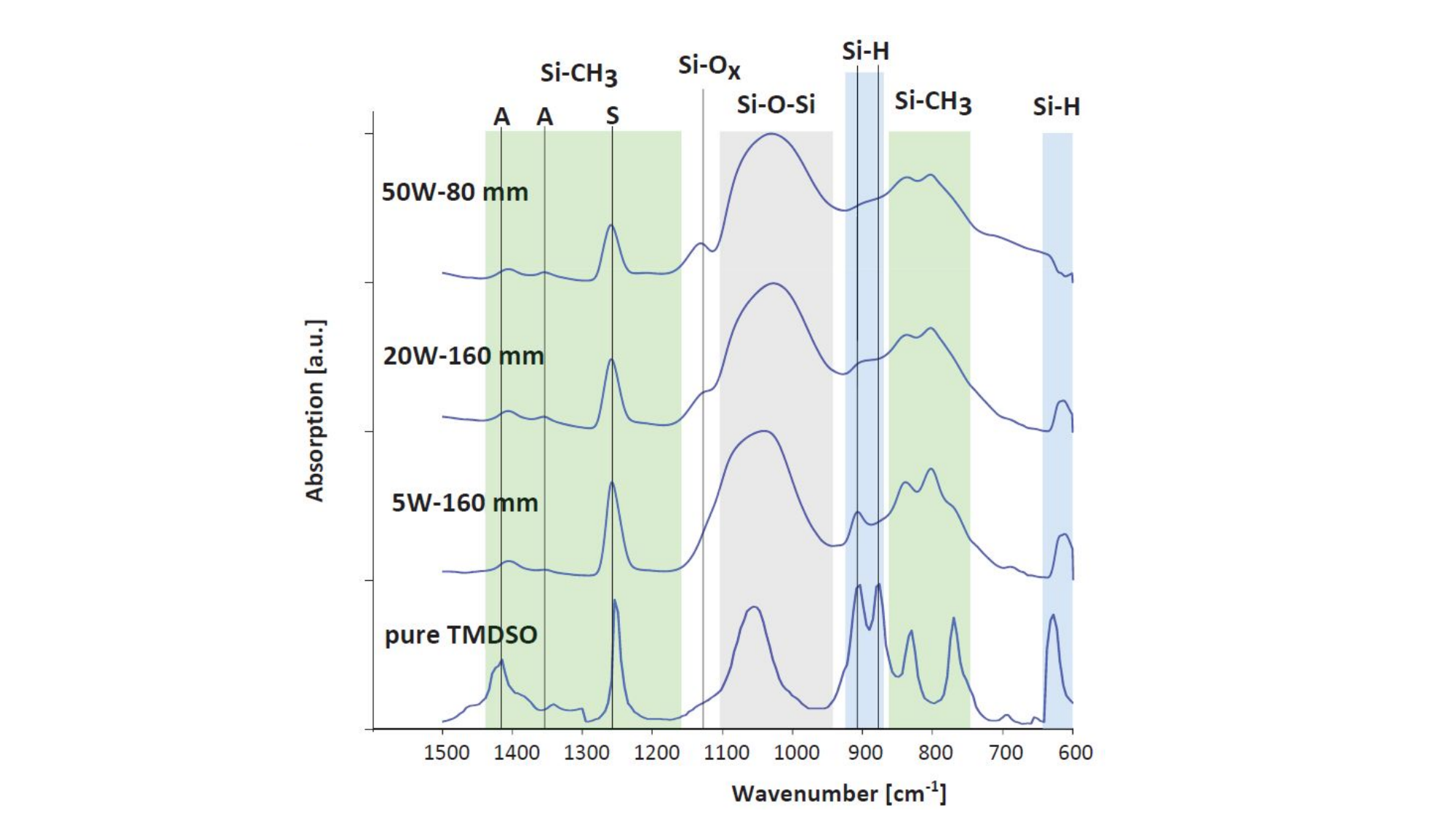

Supplement: Supplementary file 1 [file molecules-26-05621-s001.zip › Figure S1.pptx]

## Slide 1
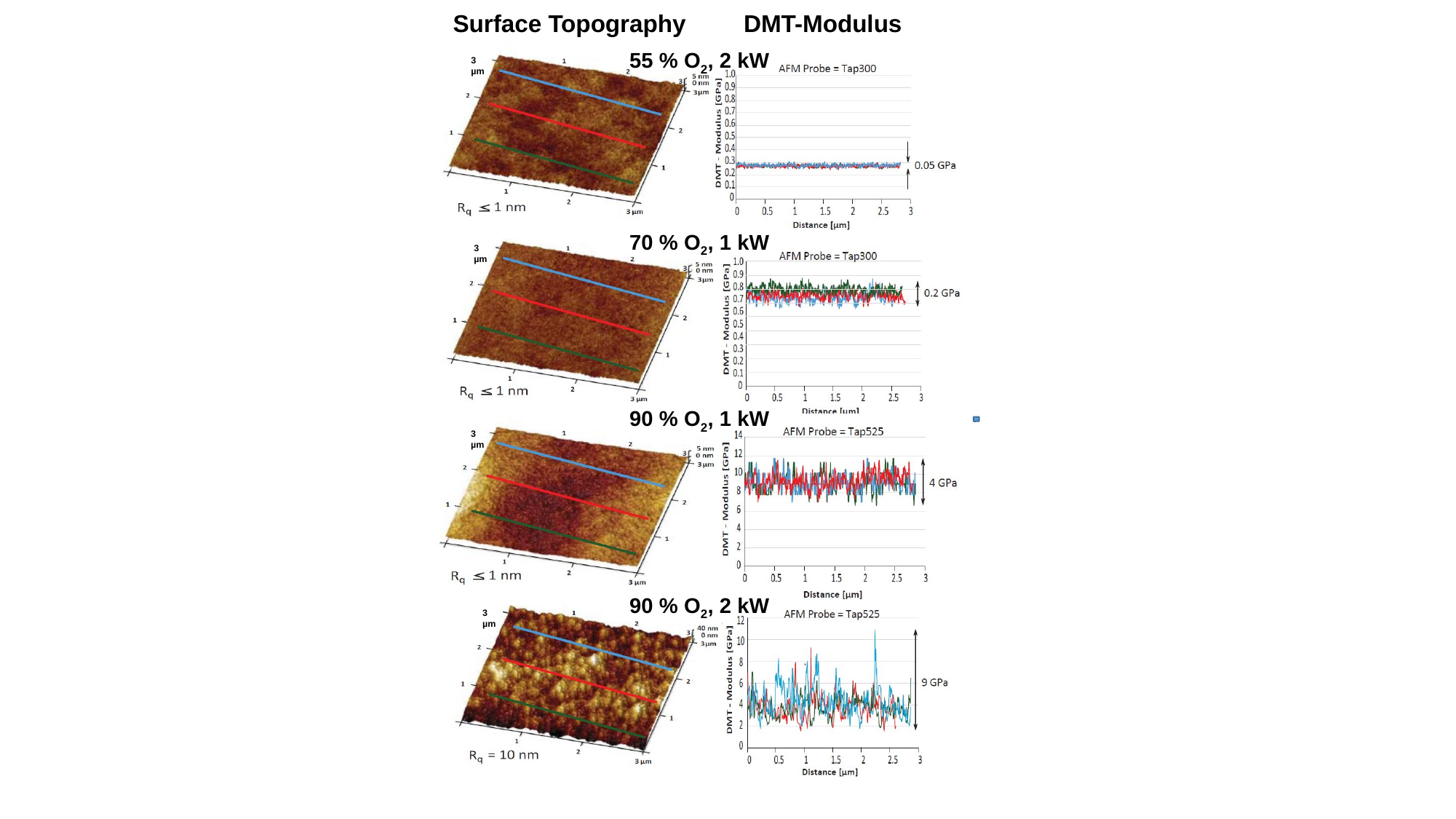

Surface Topography
DMT-Modulus
55 % O2, 2 kW
3 µm
70 % O2, 1 kW
3 µm
90 % O2, 1 kW
3 µm
90 % O2, 2 kW
3 µm

Supplement: Supplementary file 1 [file molecules-26-05621-s001.zip › Figure S2.pptx]

## Slide 1
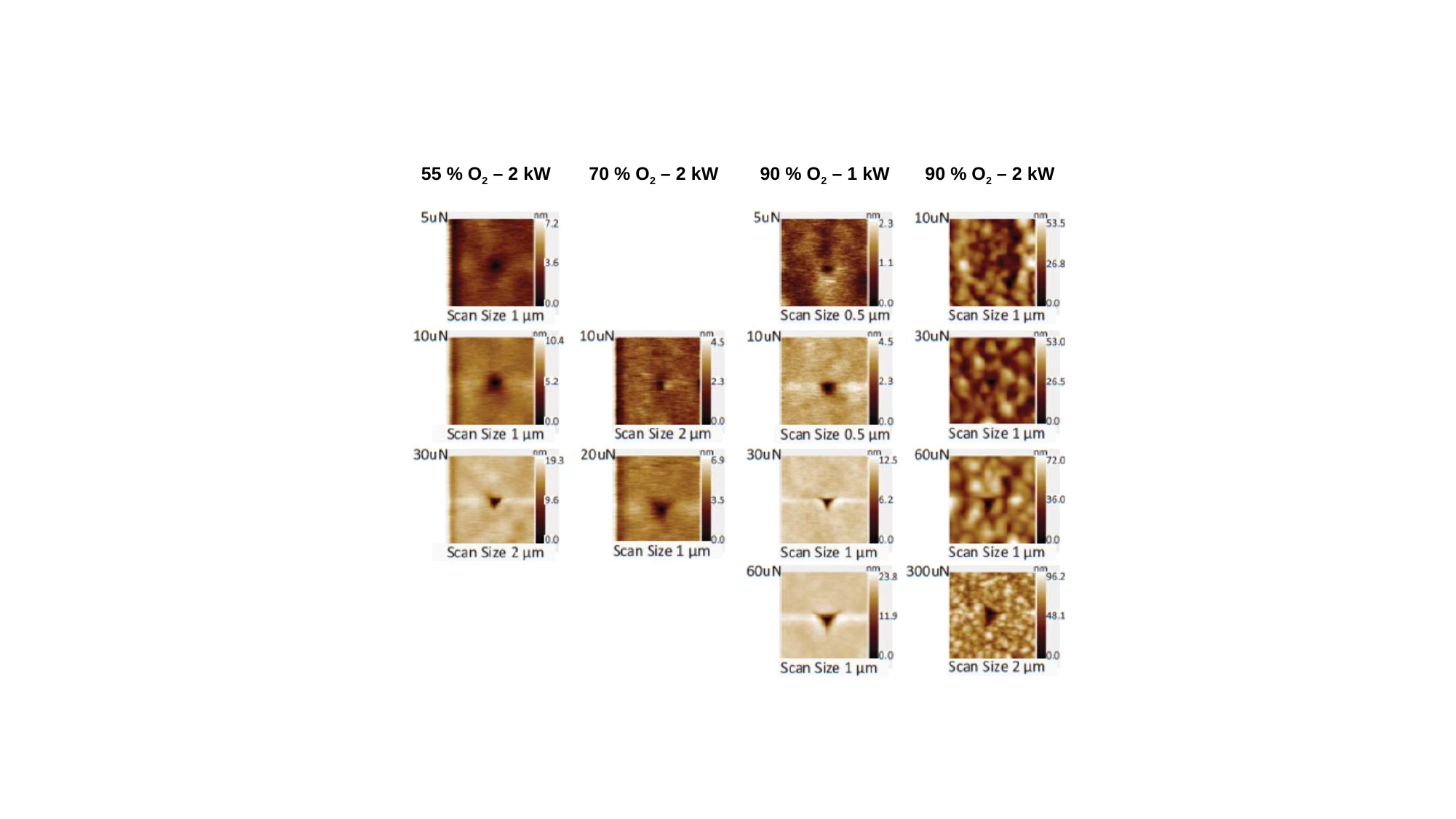

90 % O2 – 2 kW
90 % O2 – 1 kW
55 % O2 – 2 kW
70 % O2 – 2 kW

Supplement: Supplementary file 1 [file molecules-26-05621-s001.zip › Figure S3.pptx]

## Slide 1
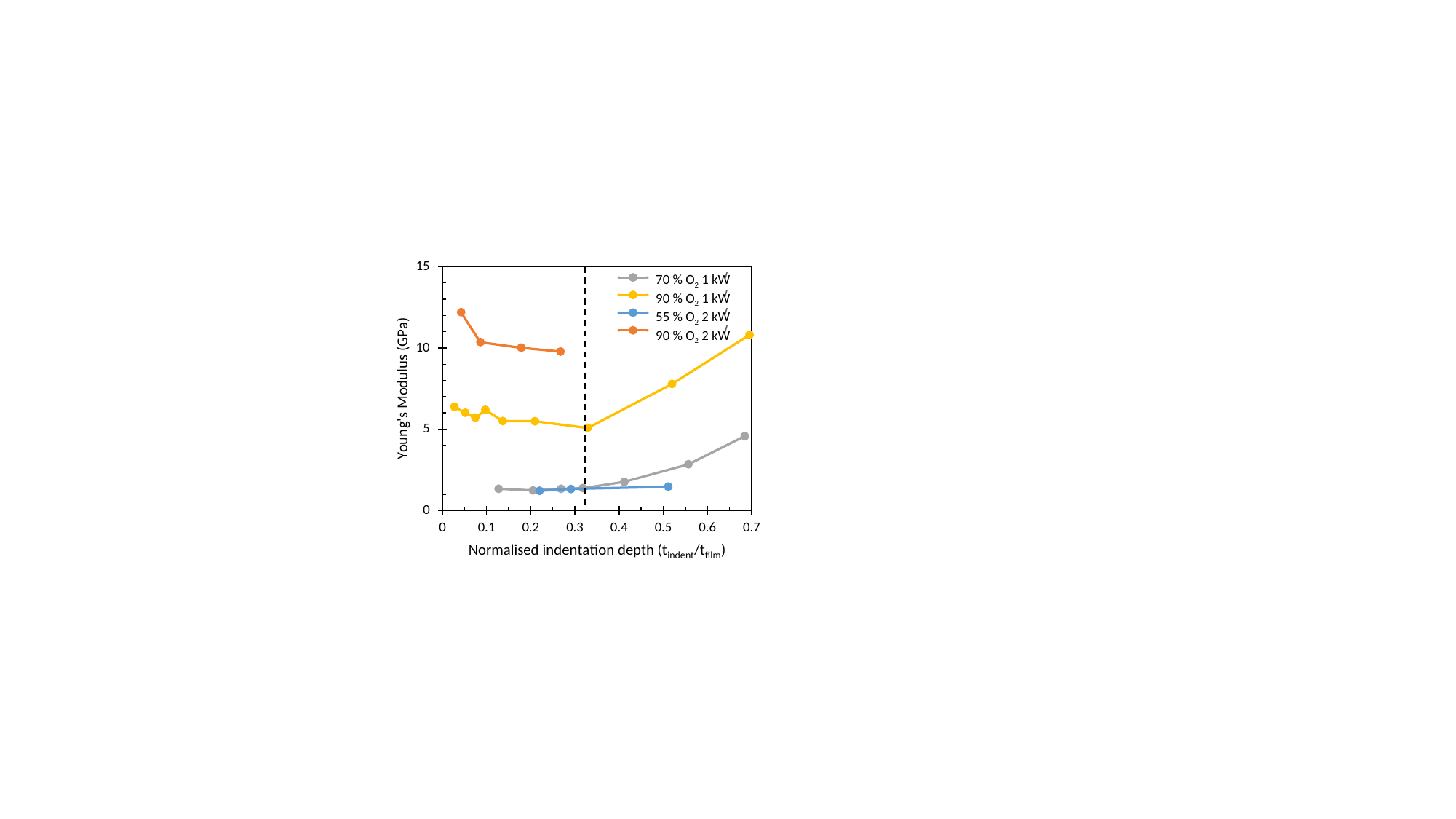

70 % O2 1 kW
90 % O2 1 kW
55 % O2 2 kW
90 % O2 2 kW

Supplement: Supplementary file 1 [file molecules-26-05621-s001.zip › Figure S4.pptx]
